# Conservation Genetics and Genomics

**DOI:** 10.3390/genes11030318

**Published:** 2020-03-17

**Authors:** Michael Russello, George Amato, Robert DeSalle, Michael Knapp

**Affiliations:** 1 Department of Biology, The University of British Columbia, 3247 University Way, FIP346, Kelowna, BC V1V 1V7, Canada; michael.russello@ubc.ca; 2 Conservation Genomics, Sackler Institute for Comparative Genomics, American Museum of Natural History, New York, NY 10024-5102, USA; desalle@amnh.org; 3 Department of Anatomy, University of Otago, Dunedin 9054, New Zealand; michael.knapp@otago.ac.nz

For more than thirty years, methods and theories from evolutionary biology, phylogenetics, population genetics and molecular biology have been used by conservation biologists to better understand threats to endangered species due to anthropogenic changes. Commonly described as Conservation Genetics, the scope of research has included investigating effects of habitat fragmentation and over-harvesting on small populations, barriers to natural gene flow, uncertainty about units of conservation due to unresolved taxonomies and cryptic species, illegal and commercial trade in wildlife, and molecular ecology of threatened populations and species [1].

Advances in genomic tools, along with demonstration of their applicability to non-invasive sampling approaches often necessary for at-risk species, have greatly expanded the purview and value of this field of study [2]. This special issue of *Genes* on “Conservation Genetics and Genomics” features 14 original research articles harnessing the data collection and analytical approaches of population genomics, phylogenomics, and metagenomics to address questions of conservation concern. Traditional approaches using neutral genetic markers now take advantage of vastly expanded data sets of single nucleotide polymorphisms (SNPs) discovered in a variety of ways, including using restriction site-associated DNA sequencing (RAD Seq) and reference genome mining [3]. Whole-genome sequencing has enabled elucidation of genome architecture, and exploration of the influences of genome-wide patterns and locus-specific effects of relevance to species of conservation concern [4,5,6]. Likewise, coalescent-based approaches applied to nuclear and organellar genomic data allow for more detailed approximations of recent and more distant evolutionary histories for endangered taxa of interest [7,8]. In other cases, broader phylogenetic understanding of a group can directly inform different kinds of conservation practice and measures [4,9]. Expanded analyses of historical and ancient DNA now afford novel opportunities for better understanding relevant processes, including contemporary and past hybridization [10,11,12]. In addition, metagenomics and environmental (e)DNA allow for broader access to genetically sample ecosystems in new and rapid ways [13,14,15]. Importantly, genomic technologies have opened avenues of research into genetic rescue and restoration, contributing to the field of de-extinction [16]. As extinction and habitat destruction become increasingly acute, expanding the technical approaches for understanding the genetics of endangered species is one of many essential aspects of conservation biology. We hope this special issue affords scientists involved in active research in the area a valuable update on genomics-focused conservation efforts and stimulus for continued interest in this crisis discipline.
